# Degradation of metaldehyde in water by nanoparticle catalysts and powdered activated carbon

**DOI:** 10.1007/s11356-017-9249-1

**Published:** 2017-06-14

**Authors:** Zhuojun Li, Jong Kyu Kim, Vrushali Chaudhari, Suseeladevi Mayadevi, Luiza C. Campos

**Affiliations:** 1 Department of Civil, Environmental and Geomatic Engineering, University College London, Gower Street, London, WC1E 6BT UK; 2 Kyungnam University, Gyeongsangnam-do Massanhappo-gu South Gyeongsang Daehakro 7, Changwon, South Korea; 3 Chemical Engineering & Process Development Division, CSIR-National Chemical Laboratory, Pune, Maharashtra 411 008 India

**Keywords:** Metaldehyde, Powdered activated carbon, Photocatalysts, Adsorption, Photocatalysis

## Abstract

Metaldehyde, an organic pesticide widely used in the UK, has been detected in drinking water in the UK with a low concentration (<1 μg L^−1^) which is still above the European and UK standard requirements. This paper investigates the efficiency of four materials: powdered activated carbon (PAC) and carbon-doped titanium dioxide nanocatalyst with different concentrations of carbon (C-1.5, C-40, and C-80) for metaldehyde removal from aqueous solutions by adsorption and oxidation via photocatalysis. PAC was found to be the most effective material which showed almost over 90% removal. Adsorption data were well fitted to the Langmuir isotherm model, giving a *q*_*m*_ (maximum/saturation adsorption capacity) value of 32.258 mg g^−1^ and a *K*_*L*_ (Langmuir constant) value of 2.013 L mg^−1^. In terms of kinetic study, adsorption of metaldehyde by PAC fitted well with a pseudo-second-order equation, giving the adsorption rate constant *k*_2_ value of 0.023 g mg^−1^ min^−1^, implying rapid adsorption. The nanocatalysts were much less effective in oxidising metaldehyde than PAC with the same metaldehyde concentration and 0.2 g L^−1^ loading concentration of materials under UV light; the maximum removal achieved by carbon-doped titanium dioxide (C-1.5) nanocatalyst was around 15% for a 7.5 ppm metaldehyde solution.

**Electronic supplementary material:**

The online version of this article (doi:10.1007/s11356-017-9249-1) contains supplementary material, which is available to authorized users.

**Graphical abstract:**
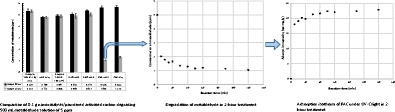
ᅟ

## Introduction

Metaldehyde, which has been reported by the UK Environment Agency, is an organic compound used as pesticide targeting slugs, snails, and other molluscs and is widely used in agriculture (UK Environment Agency 2009). There are growing concerns that relatively high levels of metaldehyde have been detected in surface water. In fact, it is reported that trace amounts of metaldehyde have been found in treated drinking water in the UK with concentrations as high as 1 μg L^−1^ which is above the European and UK standards of 0.1 μg L^−1^ (Water UK 2013). The common treatment designed to remove pesticides from water by adsorption onto granular activated carbon (GAC) or by other processes involving chlorine or ozone was proven to be ineffective in removing metaldehyde (Water UK 2013).

There are a number of studies investigating new methods to remove metaldehyde from water. For example, using ultraviolet (UV) irradiation to activate a number of different chemicals such as TiO_2_, H_2_O_2_ for the degradation of the organic pollutant to CO_2_ and H_2_O (Autin et al. 2012), and a dual-stage method of using catalyst and ion-exchange resin as adsorbent to remove metaldehyde (Tao and Fletcher 2016). However, a more cost-effective method is still needed to solve this issue.

The reason why metaldehyde is not effectively degraded by GAC could be that the particle size and surface area of GAC are not suitable for the removal of metaldehyde. Therefore, one alternative approach would be to use powdered activated carbon (PAC) which has smaller particle sizes than traditional GAC, thereby providing more pore and surface space for adsorption. Another approach that has potential to remove organic pollutants would be advanced oxidation processes (AOPs), which applies UV irradiation to catalysts such as TiO_2_ to produce ·OH radicals and attack organic molecules (Zhang et al. 2012; Doria et al. 2013; Kim et al. 2013; Ribeiro et al. 2015). TiO_2_ as a widely studied photocatalyst has shown its potential for removing organic pollutants from water. For instance, Chung and Chen (2009) found that azo dye reactive violet 5 was successfully removed by TiO_2_ photocatalysis; Lin et al. (2011) studied the degradation of benzylparaben by UV/TiO_2_.

This study investigated the effectiveness of using three novel nanocatalysts, i.e., C-doped TiO_2_ with a carbon content of 1.5, 40, and 80% under UV-C light to remove metaldehyde from aqueous solution. The photocatalytic activity of TiO_2_ for degradation of dilute pollutants is known to be enhanced by addition of small amounts of absorbents, in particular, activated carbon and zeolites (Agrios and Pichat 2005). The adsorbent-catalyst system interacts synergistically leading to a higher performance of degradation of metaldehyde in water. The National Chemical Laboratory (NCL) had developed carbon from cheap agro-wastes and used it in TiO_2_ synthesis which showed high performance for the degradation of certain dyes. The efficiency of these catalysts for metaldehyde degradation has been compared with that of PAC in this work. The specific objectives of this study were (1) to determine the effect of initial metaldehyde concentration on degradation; (2) to find out the effectiveness of PAC and the novel catalysts on metaldehyde degradation; (3) to check the effect of UV-C light on metaldehyde degradation; and (4) to analyse the adsorption and kinetics of metaldehyde degradation.

## Materials and methods

### Specification of PAC and synthesis of nanocatalysts

Commercial PAC (charcoal, decolorizing powder activated) used in the work was Darco G60, manufactured by the British Drug Houses (BDH) laboratory supplies. Its carbon source is charcoal, and it is certified for a maximum use level of 250 mg/L (National Sanitation Foundation 2015). In addition, the following C-doped TiO_2_ nanocatalysts were used:

- Cetyltrimethylammonium bromide (CTAB)-modified carbon-doped titanium dioxide (C-doped TiO_2_) nanocatalyst with 1.5% carbon, 98.5% TiO_2_ (C-1.5)
- CTAB-modified C-doped TiO_2_ nanocatalyst with 40% carbon, 60% TiO_2_ (C-40)
- CTAB-modified C-doped TiO_2_ nanocatalyst with 80% carbon, 20% TiO_2_ (C-80)

The C-doped TiO_2_ nanocatalysts were provided by the National Chemical Laboratory (NCL) in India. The C-doped TiO_2_ catalyst (C-1.5) was made using 7.372 g titanium butoxide, 33.818 g isopropanol, 7 mL H_2_O, 0.5 g urea, 0.03 g carbon made from sugar cane leaf agro-waste, and 5 g CTAB. The procedure of synthesis was as follows: titanium butoxide and isopropanol were added together and stirred for 0.5 h, and CTAB was added to H_2_O and isopropanol and mixed well. After that, urea was dissolved in the mixture and then carbon was added. This mixture then was added into the previous butoxide solution and stirred for 24 h at room temperature. Then, the mixture was dried at 80 °C for 5 h and lastly calcined at 300 °C for 3 h. C-40 and C-80 nanocatalysts were made by the same procedure but, this time, varying the amounts of carbon and titanium butoxide.

The characterization of PAC was determined by the Micromeritics Instrument Corporation in Korea using AutoPore IV 9500 V1.07. The scanning electron microscope (SEM) images of PAC and C-doped TiO_2_ (C-1.5) were captured with an accelerating voltage of 20 kV. The characterization of C-doped TiO_2_ (C-1.5) was determined by the National Chemical Laboratory.

Other materials included metaldehyde, HPLC-grade methanol, and HPLC-grade dichloromethane (DCM). One gram of solid metaldehyde PESTANAL was purchased from Sigma-Aldrich.

### Preparation of metaldehyde standard solutions

During the whole experiment and sample preparation, Millipore water was used. This was because deionised water could have a high organic content and may react with ·OH radicals that are produced by photocatalysis during the reaction and thereby affect the GC-MS analysis. Metaldehyde stock solution was prepared by the reference method from the UK Environment Agency (UK Environment Agency 2009). Metaldehyde solid (0.1 g) was added into 100 mL methanol to make 1000 ppm metaldehyde stock solution. Metaldehyde stock solutions could be stored between 1 and 10 °C for up to 1 year. For each photocatalytic experiment, a different amount of stock solution was diluted by Millipore water to 1000 mL to prepare sample solutions with different metaldehyde concentrations. The studied range of metaldehyde concentrations was from 0.1 to 15 ppm.

### Analytical methods

Metaldehyde was analysed by gas chromatography (Perkin Elmer precisely Clarus 500) with mass spectrometry (GC-MS). All samples of metaldehyde solution taken from the photoreactor were filtered using a MILLEX 0.22-μm syringe-driven membrane filter unit (manufactured by Millipore Express) before passing through a pre-conditioned solid phase extraction column (SDB SPE disposable extraction columns, 3 mL, 200 mg 126 BAKERBOND™ spe). After extracting metaldehyde from the aqueous phase to the organic phase (in DCM), the sample was then transferred to the autosampler. All samples were prepared as triplicates, and each sample was injected three times by the autosampler to ensure repeatability and minimise instrumental error. Before injection of the samples, pure DCM was first injected to ensure samples were not contaminated from previous use of the GC-MS (Fig. S1). The detection of metaldehyde by GC-MS was made using the parameters in Table S1 in the supplementary materials. The solid phase extraction method is described in the Appendix, and the recovery rates of metaldehyde for each set of experiments from the aqueous phase to the organic phase are listed in Table S2 in the supplementary materials.

**Fig. 1:**
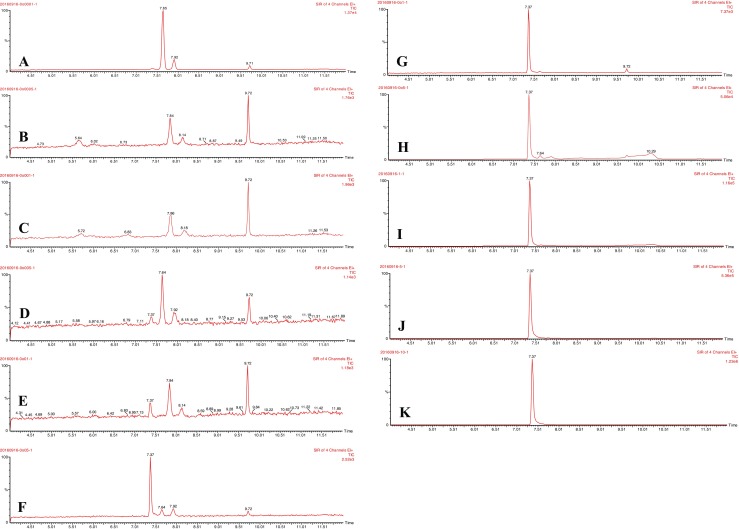
Detection of metaldehyde by GC-MS from 0.1 ppb to 10 ppm: **a** 0.1 ppb, **b** 0.5 ppb, and **c** 1 ppb show no peak of metaldehyde; **d** 5 ppb and **e** 10 ppb show low peaks of metaldehyde; **f** 50 ppb, **g** 0.1 ppm, **h** 0.5 ppm, **i** 1 ppm, **j** 5 ppm, and **k** 10 ppm show distinctive peaks of metaldehyde

The detection limit of metaldehyde by GC-MS was tested by preparing a range of metaldehyde solutions in DCM from 0.1 ppb to 10 ppm. From Fig. 1 (chromatograms showing peaks of metaldehyde and DCM), it was suggested that the detection limit was between 1 and 5 ppb. For metaldehyde concentrations equal or higher than 5 ppb, the peak of metaldehyde at 7.37 min could be detected, together with the peak of DCM at 9.72 min, and for concentrations equal or higher than 50 ppb, the peaks of metaldehyde were distinctive with low peaks of DCM presented at 9.72 min. As the concentration of metaldehyde increased, the peaks of metaldehyde became more distinctive while the peaks of DCM became less distinguishable, especially when concentrations of metaldehyde were higher than 0.5 ppm. For metaldehyde concentration below 50 ppb, there were a few peaks at 7.64, 7.84, 7.90, and 8.14 min which were worth noting. From Fig. S1 and Fig. 1, these peaks can be identified as decomposed components of DCM from the heat of GC-MS when running with injection temperature of 180 °C and oven temperature of 150 °C; because DCM is extremely volatile, it would partially decompose on heating and might produce vapours of HCl, CO, and COCl_2_ (International Labour Organization 2012).

### Batch photoreactor system

All experiments were performed in a batch reactor using a photoreactor with an ultraviolet (UV) lamp as the source of radiation. This batch photoreactor system followed the one proposed by Kim et al. (2013). The photoreactor was a rectangular box made from stainless steel with four valves installed at the bottom and top (Figs. S2 and S3 in the supplementary materials). The UV lamp used was a UV-C medium-pressure mercury-vapour Philips lamp, of 11 W, 240 V, and 254 nm wavelength, made in Holland. The light density of this lamp was 35 μmol m^−2^ s^−1^ (11.4 W m^−2^), measured by a lux metre (Apogee, model MQ-100, serial number 1514, made in the USA). The UV-C lamp was inserted vertically and mounted from the top of the reactor which enabled it to make contact with solution inside. The reactor was surrounded by a water cooling jacket to prevent the sample solution from being heated by the UV-C lamp during the course of the reaction. Hence, a constant temperature of around 25 °C (room temperature) was maintained. The reactor was connected to an air source from the air tap to ensure that PAC or nanocatalysts were well mixed and evenly distributed in the solution from bottom to top inside of the reactor. The air supply was maintained at 1 cm^3^/min through an air flow metre manufactured by CT Platon. In addition, a magnetic stirrer was placed inside the reactor to stir the sample solution and ensure that PAC or nanocatalysts were in contact with the solution. For all experiments, the volume of the metaldehyde solution was 500 mL, the loading concentration of PAC or catalyst was 0.2 g L^−1^, and reaction time was 2 h.

At first, a set of experiments were carried out to compare the efficiency of all the materials in removing 5 ppm metaldehyde solution under different conditions: C-doped TiO_2_ (C-1.5), UV-C light only, C-doped TiO_2_ (C-1.5) with UV-C light, C-40 with UV-C light, C-80 with UV-C light, PAC with UV-C light, and PAC in the dark. After that, using the most effective material (PAC) under UV-C light, the adsorption isotherm was determined by using 0.1 g PAC to degrade 500 mL metaldehyde solutions with different concentrations that ranged from 0.1 to 10 ppm. To compare with adsorption of metaldehyde by PAC, a set of experiments were carried out to analyse photocatalysis of metaldehyde (concentration range from 0.1 to 12 ppm) by C-doped TiO_2_ (C-1.5) nanocatalyst.

## Results

### Characterization of PAC and nanocatalysts

**Fig. 2:**
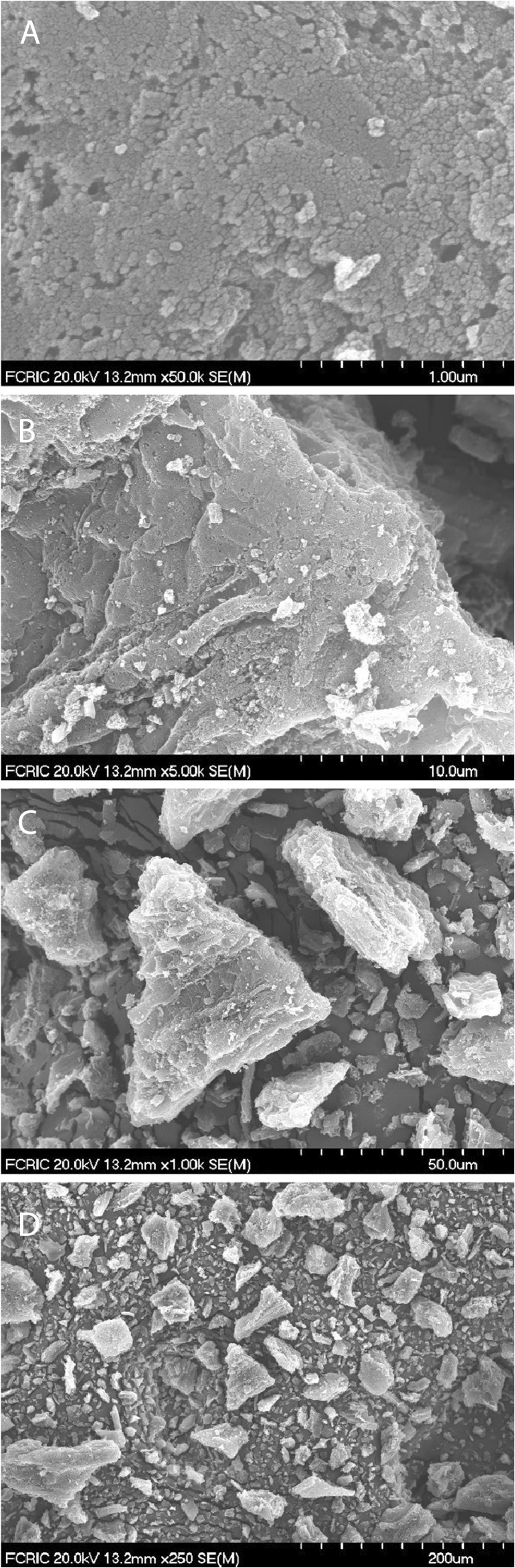
SEM images of powdered activated carbon (PAC). **a** (At 1 μm scale) the surface porous structure of carbon crystal, **b** (At 10 μm scale) the edges of carbon crystals where potential adsorption takes place. **c** (At 50 μm scale) the angular shape of carbon crystals with many edges. **d** (At 200 μm scale) an overview of carbon crystals with different sizes

Figure 2 presents the scanning electron microscope (SEM) image of PAC which shows the structure and surface characterization of the PAC. The images illustrate that the average size of carbon particle is approximately 20 nm, and these particles have aggregated together and formed angular-shaped clusters with an average size of 25 μm. The porosity is 17.78%. From the SEM images, the surface of the clusters is flat, rough, and porous.

**Fig. 3:**
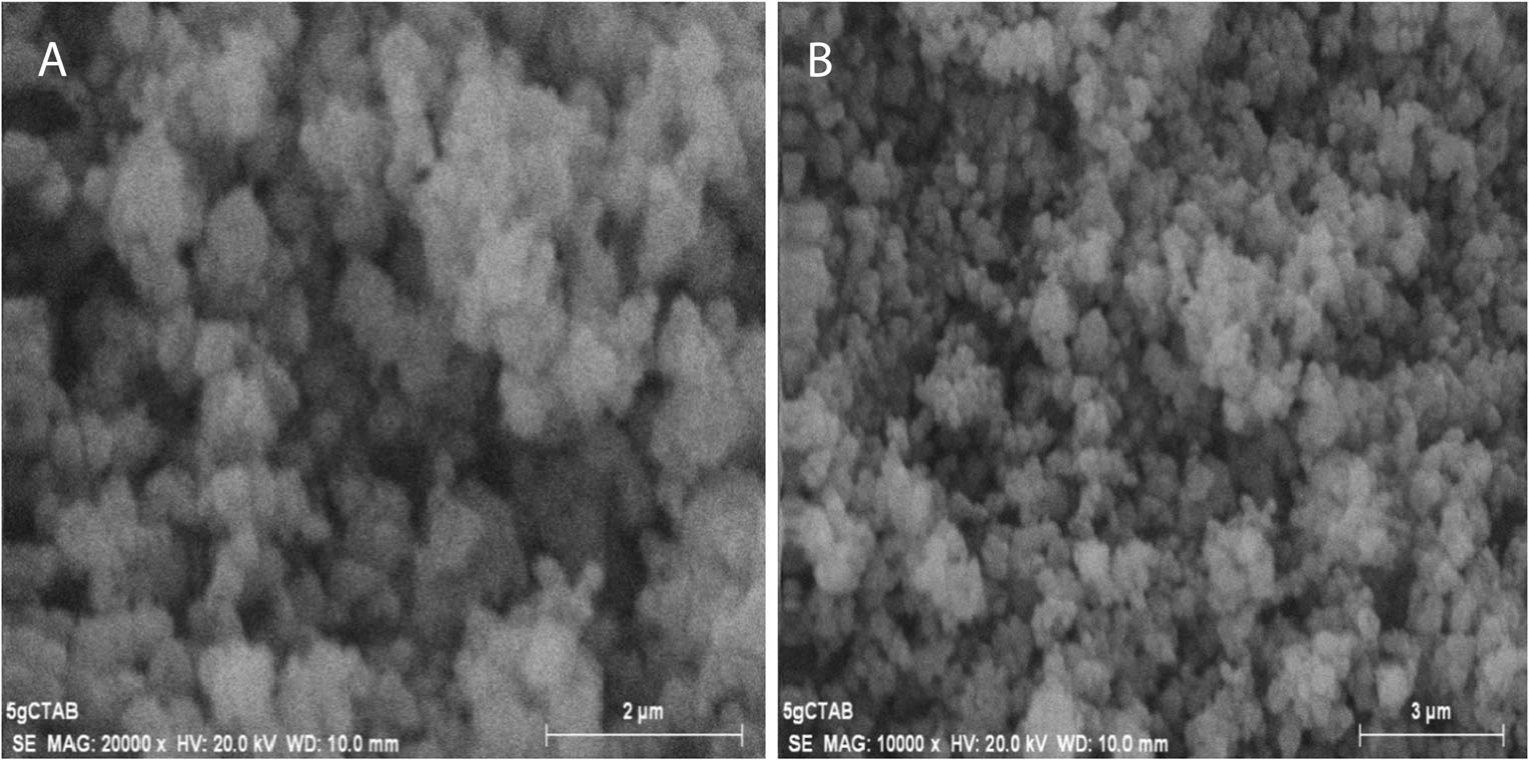
SEM images of C-doped TiO_2_ (C-1.5) nanocatalyst. **a** (At 2 μm scale) The round shape and porous structure of the crystals. **b** (At 3 μm scale) The shape and size of crystals are more or less uniform

**Fig. 4:**
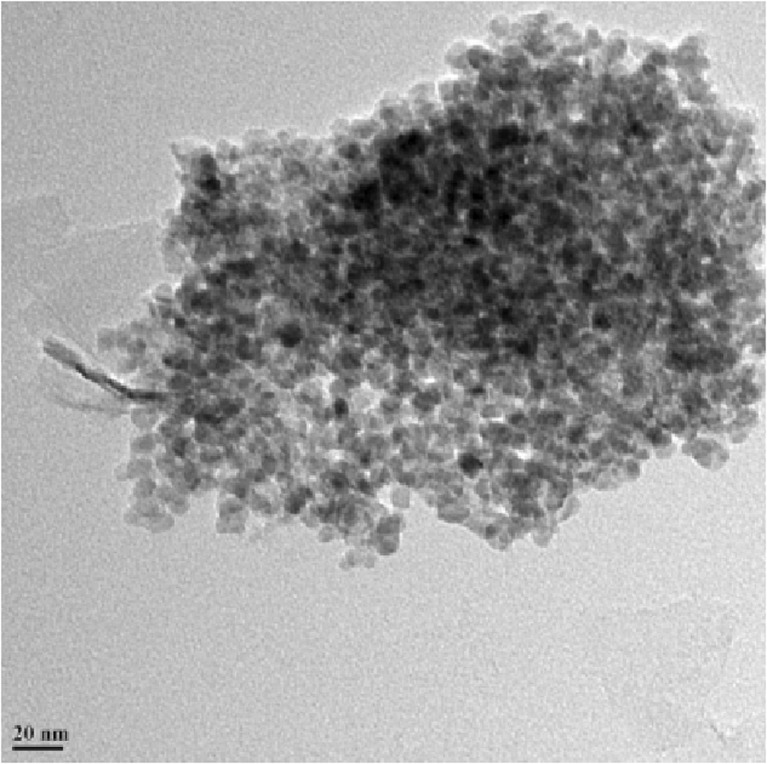
TEM image of C-doped TiO_2_ (C-1.5) nanocatalyst: carbon particles in *black colour* can be seen on the surface of TiO_2_ particles in *white colour*

Figure 3 shows the SEM images of C-doped TiO_2_ (C-1.5). As titanium butoxide solution was added to pseudo-homogeneous solution containing carbon particles (made from sugar cane leaf agro-waste) under rigorous stirring, it is possible that TiO_2_ would be formed with the carbon particles decorating it. However, the TEM image presented by Fig. 4 shows the presence of carbon on the TiO_2_ surface; therefore, it is not considered as TiO_2_-decorated carbon particles. The crystal size of the nanoparticles is around 10 nm. Compared to the PAC, the shape of the nanocatalyst particles is more rounded. These particles have agglomerated together forming clusters, which appear to have a rough, porous surface. The surface area of C-doped TiO_2_ nanocatalyst is 115.06 m^2^ g^−1^, total pore volume is 0.3349 cm^3^ g^−1^, and average pore diameter is 105.8 A°. The characterizations of PAC and C-doped TiO_2_ (C-1.5) nanocatalyst are presented in Table 1. The characterizations of C-40 and C-80 nanocatalysts are at different stages, and therefore are not shown here.

**Table 1:** Characterization of PAC and C-doped TiO_2_ (C-1.5)

Material	PAC	C-doped TiO_2_ (C-1.5)
Total intrusion volume (mL g^−1^)	0.4192	0.3349
Crystal size (nm)	20	12
Average pore diameter (4 V/A) (nm)	619	1058
Surface area (m^2^ g^−1^)	962	115

### Effect of UV-C light and increasing carbon content of nanocatalysts

A set of experiments were carried out to determine the role of UV-C, PAC, C-doped TiO_2_ (C-1.5), C-40, and C-80 nanocatalysts on metaldehyde degradation under controlled conditions, including presence of PAC/nanocatalysts only, UV-C light only, and both PAC/nanocatalysts and UV-C. The prepared metaldehyde solution concentration for this experiment before all treatments was 5 ppm, while the nanocatalyst loading concentration was 0.2 g L^−1^.

**Fig. 5:**
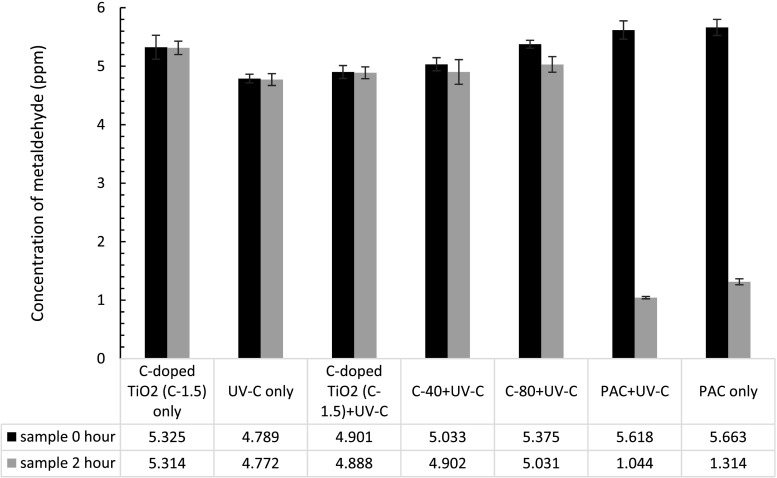
Concentrations of metaldehyde solution before and after different treatments: UV-C light has a wavelength of 254 nm; each *bar* represents nine experimental data (triplicates samples and three injections into GC-MS; *error bars* showing standard errors

Figure 5 shows the concentration of metaldehyde after each treatment. All data has a relative standard deviation (RSD) less than 6%, suggesting quite high precision and accuracy from experiment performance and instrumental analysis. There was no significant metaldehyde degradation by the nanocatalysts alone, UV-C light alone, and nanocatalysts with UV-C light (Table 2). An ANOVA single-factor statistic test was performed to determine whether there was a significant difference (*p* < 0.05) before and after different treatment methods. The treatment methods incorporating C-80 with UV-C, PAC with UV-C, and PAC only (marked with asterisks) show significant differences of metaldehyde concentrations before and after each treatment (*p* < 0.05).

**Table 2:** Percentage removal of metaldehyde by the various materials

Treatment	Metaldehyde removal (%)
C-doped TiO_2_ (C-1.5) only	0
UV-C only	1 ± 1
C-doped TiO_2_ (C-1.5) + UV-C	2 ± 2
C-40 + UV-C	4 ± 4
C-80 + UV-C*	6 ± 4
PAC + UV-C*	81 ± 1
PAC only*	77 ± 1

The increased carbon content of nanocatalysts from 1.5 to 80% only slightly increased the removal of metaldehyde by 2 and 4%, respectively. On the other hand, PAC alone removed a substantial amount of metaldehyde by 77% which confirmed that adsorption is one of the favourable removal mechanisms for metaldehyde. Busquets et al. (2014) had similar findings with mesoporous phenolic carbon which demonstrated effective degradation of metaldehyde with an adsorption capacity of 76 mg g^−1^ for 64 ppm metaldehyde. Interestingly, degradation of metaldehyde by PAC under UV-C light was slightly more effective (by 4%) than PAC alone under dark conditions. This could indicate that metaldehyde can be more effectively degraded by a combination of adsorption and photolysis which could be a promising technique in water and wastewater treatment. Similarly, there were studies implying combination of UV light and GAC can increase degradation efficiency by more than 50%, regarding removal of total solid concentration, total volatile solids, and biochemical oxygen demand of wastewater (Asha et al. 2015).

### Degradation of metaldehyde using PAC

**Fig. 6:**
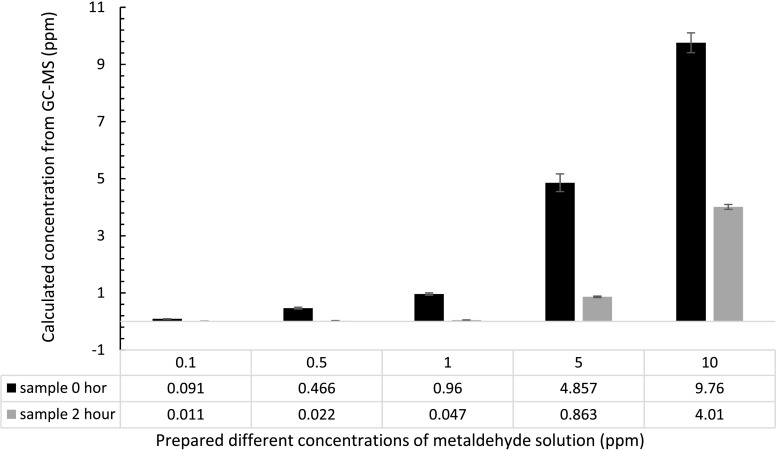
Concentrations of metaldehyde before and after 2-h treatment by 0.1 g PAC: UV-C light has a wavelength of 254 nm; each bar represents nine experimental data (triplicates samples and three injections into GC-MS; *error bars* showing standard errors

PAC (0.1 g) was used in this set of experiments with different prepared initial concentrations of metaldehyde samples at 0.1, 0.5, 1, 5, and 10 ppm under UV-C light for the 2-h treatment. Figure 6 compares the concentration of metaldehyde before and after treatment by PAC. PAC effectively removed metaldehyde from the solution, especially for lower initial concentrations of 0.1, 0.5, and 1 ppm (*p* < 0.05).

**Fig. 7:**
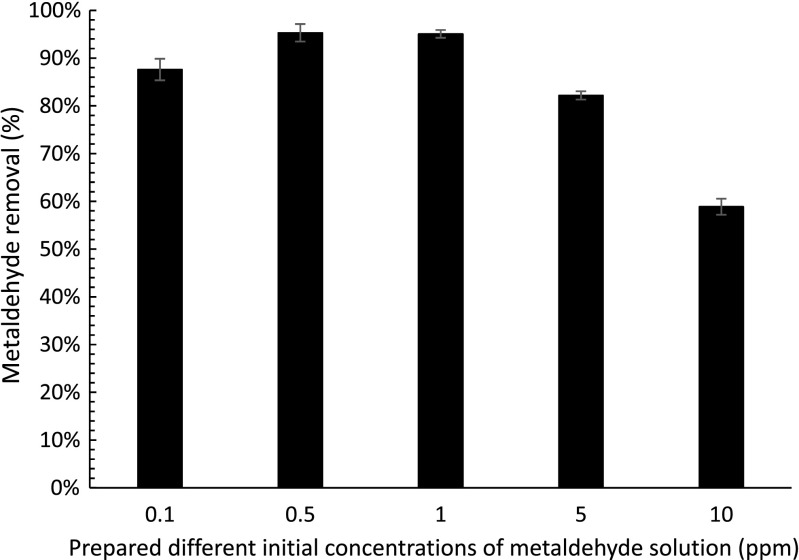
Percentage removal of metaldehyde solution using PAC: UV-C light has a wavelength of 254 nm; each *bar* represents nine experimental data (triplicates samples and three injections into GC-MS; *error bars* showing standard errors

Figure 7 shows that the removal of metaldehyde decreases as the initial concentration of metaldehyde increases. It can be seen that the removal of metaldehyde at lower concentrations were 88, 95, 95, 82, and 59% for 0.1, 0.5, 1, 5, and 10 ppm, respectively. The removal of metaldehyde was slightly lower at 0.1 ppm than those at 0.5 and 1 ppm. This is because adsorption at low concentration indicates there is a considerable amount of adsorption sites but only with a small amount adsorbate, and when exceeding a certain ratio of adsorbent and adsorbate, adsorption slows down (Nandi et al. 2008). Therefore, the reaction is slower and more adsorption time is needed for more effective removal of metaldehyde.

### Degradation of metaldehyde using C-doped TiO_2_ (C-1.5) nanocatalyst

**Fig. 8:**
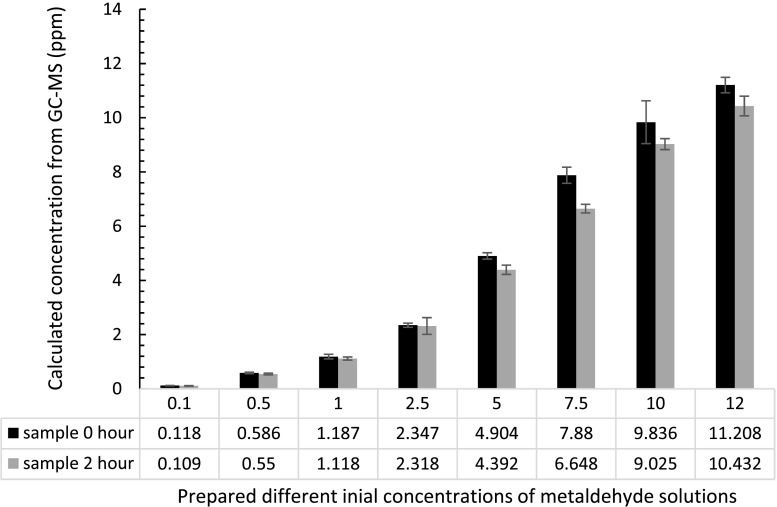
Concentrations of metaldehyde before and after 2-h treatment by 0.1 g C-doped TiO_2_ (C-1.5) nanocatalyst: UV-C light has a wavelength of 254 nm; each *bar* represents nine experimental data (triplicates samples and three injections into GC-MS; *error bars* showing standard errors

C-doped TiO_2_ (C-1.5) nanocatalyst is considered not effective regarding degradation of metaldehyde in water, especially compared with PAC. Figure 8 shows the concentrations of metaldehyde in solution before and after the 2-h treatment by C-doped TiO_2_ (C-1.5) nanocatalyst under UV-C light. At higher initial concentrations of metaldehyde solutions such as 5, 7.5, 10, and 12 ppm, the degradation was slightly more significant (2–9%), compared to that at lower concentrations.

Only for prepared concentrations of metaldehyde higher than 5 ppm, there was a significant difference (*p* < 0.05) before and after treatment, suggesting there was no degradation of metaldehyde at prepared initial concentrations at 0.1, 0.5, 1, and 2.5 ppm. The degradation of metaldehyde at 7.5 and 10 ppm (Table 3) was the highest with removal of metaldehyde of 15 ± 5 and 13 ± 5%, respectively. This can be explained by the following: (1) photocatalysis reaction is slow with low concentrations of contaminants and it would require longer reaction time to effectively degrade contaminants (Dionysios et al. 2016); therefore, at lower concentrations (<5 ppm), 2 h reaction time would not be enough to effectively degrade metaldehyde, and (2) at high concentrations, the active sites on the surface of the nanocatalyst would be gradually filled by metaldehyde molecules; therefore, removal of metaldehyde would be lower.

**Table 3:** Percentage degradation of metaldehyde using 0.1 g C-doped TiO_2_ (C-1.5) under UV-C light with a wavelength of 254 nm

Prepared initial concentration (ppm)	Metaldehyde removal (%)
5	10 ± 1
7.5	15 ± 5
10	13 ± 5
12	7 ± 3

Degradation of metaldehyde by C-doped TiO_2_ was not significant compared to the values of Autin et al. (2012) who found complete degradation of 1 ppm of metaldehyde using 0.3 mM of TiO_2_ (0.024 g) with 600 mJ cm^2^ UV radiation. In our case, 0.1 g of C-doped TiO_2_ (C-1.5) cannot degrade 1 ppm metaldehyde. Therefore, this suggests that further investigation is needed to investigate the degradation using higher concentrations of the catalysts (>0.1 g) or stronger UV radiation.

## Discussion

### Fitting adsorption isotherm models for PAC

**Fig. 9:**
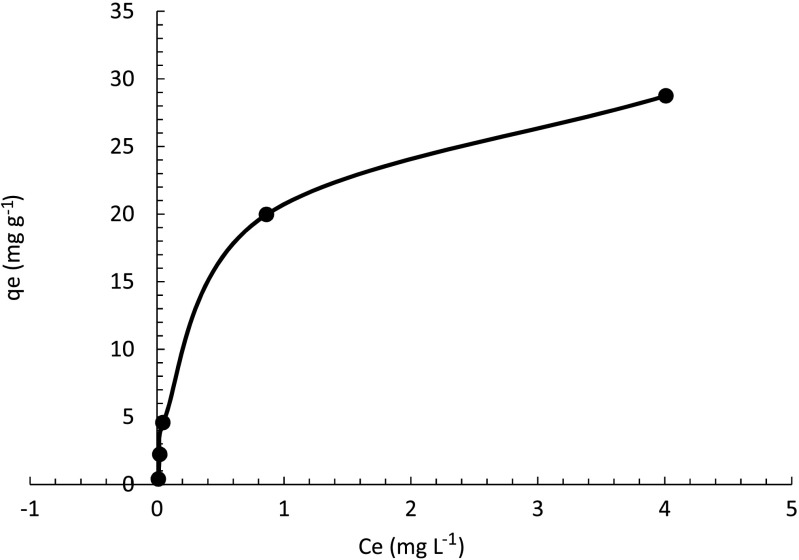
Adsorption isotherm of metaldehyde degradation by PAC

The adsorption isotherm of metaldehyde degradation by PAC under UV-C light is shown in Fig. 9. The adsorption uptake at equilibrium (*q*_*e*_: concentration of solute metaldehyde on the surface of the adsorbent PAC) can be calculated from the initial solution concentration (*C*_0_) at *t* = 0, solution concentration after 2 h of contact time (*C*_*e*_: final concentration of solution in equilibrium), and the material (PAC) loading concentration (*C*_solid_) as Eq. (1) demonstrates (Kumar et al. 2008).

1 $$ {q}_e=\frac{C_0-{C}_e}{C_{\mathrm{solid}}} $$

**Fig. 10:**
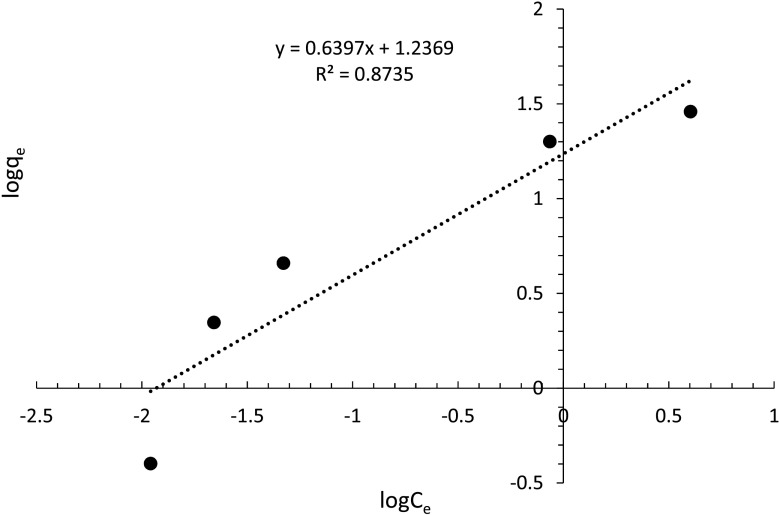
Freundlich model of metaldehyde degradation by PAC

The plot of *q*_*e*_ against *C*_*e*_ in Fig. 10 suggests that metaldehyde adsorption obeys two possible adsorption isotherm models: Freundlich model and Langmuir model.

The Freundlich model can be represented by Eq. (2) which shows the empirical relationship between *C*_*e*_ and *q*_*e*_ with two specific Freundlich constants, *K*_*F*_ (indicates adsorption capacity) and 1/*n* (indicates adsorption intensity), that are dependent on the adsorbate and adsorbent (Kumar et al. 2008).

2 $$ {q}_e={K}_F\kern.4em {C}_e^{1/ n} $$

The Freundlich model is linearized as Eq. (3) to find *K*_*F*_ and 1/*n* by linear regression (Fig. 10).

3 $$ \log {q}_e= \log {K}_F+\frac{1}{n}\kern.4em  \log {C}_e $$

Figure 10 shows that the data do not fit well with the Freundlich model (*R* ^2^ = 0.8735). The *K*_*F*_ value obtained is 0.092 (mg g^−1^)/(mg L^−1^)^1/n^, and the 1/*n* value obtained is 0.64 (*n* = 1.563). In fact, due to effective degradation of metaldehyde by PAC, the *K*_*F*_ value, as an indicator of adsorption capacity, is supposed to be larger than the obtained value from the Freundlich model. Nevertheless, the *K*_*F*_ value here is quite insignificant. From the study by Radjenovic and Medunic (2015), effective degradation gives a *K*_*F*_ value of 1.074 (mg g^−1^)/(mg L^−1^)^1/n^, and 1/*n* is an indicator of the distribution of energy sites. A high value of 1/*n* suggests high affinity between the adsorbate and the adsorbent. 1/*n* is 0.64 suggesting that 64% of the active adsorption sites have equal energy levels. Although the value of 1/*n* does fit in the beneficial adsorption (0.1 ≤ 1/*n* ≤ 1), the low 1/*n* value cannot explain the actual effective degradation of metaldehyde (Radjenovic and Medunic 2015). Therefore, the Freundlich model is suggested as not being suitable for fitting the data of metaldehyde degradation by the PAC in this study.

The Langmuir model shows the relationship between *C*_*e*_ and *q*_*e*_ with two constants, *K*_*L*_ (Langmuir constant in L mg^−1^) and *q*_*m*_ (maximum/saturation adsorption capacity in mg g^−1^) (Radjenovic and Medunic 2015), shown by Eq. (4).

4 $$ {q}_e=\frac{K_L\kern.4em {C}_e\kern.4em {q}_m}{1+{K}_L\kern.4em {C}_e} $$

**Fig. 11:**
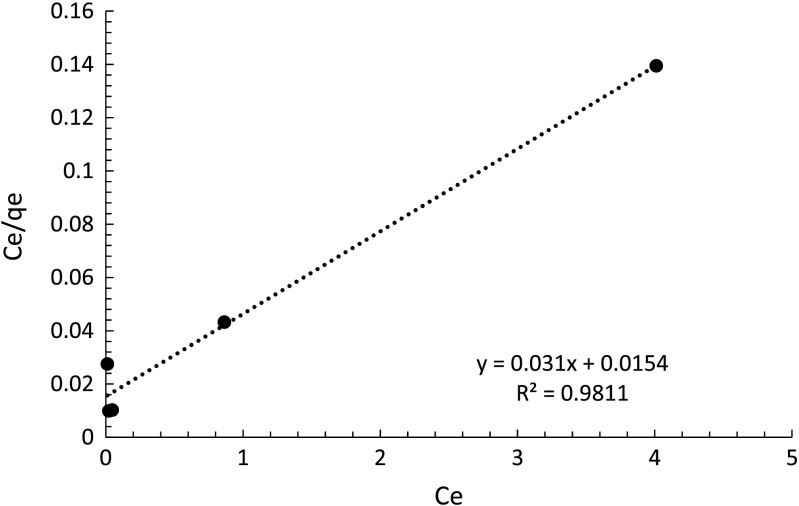
Langmuir model of metaldehyde degradation by PAC

The Langmuir model is linearized as Eq. (5), and shown by Fig. 11 so that the intercept of 1/*K*_*L*_ *q*_*m*_ and slope 1/*q*_*m*_ could be found (Radjenovic and Medunic 2015).

5 $$ \frac{C_e}{q_e}=\frac{1}{K_L\kern.4em {q}_m}+\frac{C_e}{q_m} $$

Figure 11 shows that that data fit very well with the Langmuir model (*R* ^2^ = 0.9811). The *q*_*m*_ value obtained is 32.258 mg g^−1^ and the *K*_*L*_ is 2.013 L mg^−1^. Effective degradation from the study by Radjenovic and Medunic (2015) gives *q*_*m*_ of 12.71 mg g^−1^ and *K*_*L*_ of 0.0211 L mg^−1^, and here, both values of *q*_*m*_ and *K*_*L*_ are larger than that, thereby indicating effective degradation of metaldehyde by PAC in this experiment. Based on the results of the fitting to the Langmuir isotherm, PAC has a *q*_*m*_ value of 32.258 mg g^−1^. It is worth noting that the value of *q*_*m*_ calculated in this work is much higher than the value of 12.8 mg g^−1^ (Busquets et al. 2014) obtained using industrial GAC. This is likely to be associated with the higher specific surface area of PAC used for our experiments (962 m^2^ g^−1^) compared with that used in the earlier studies (500 m^2^ g^−1^). Moreover, the sorbent used here has a higher affinity for metaldehyde, as the initial slope of its isotherm is greater than that of the GAC. Therefore, the Langmuir model is considered a better model for representing metaldehyde degradation using the PAC investigated.

### Adsorption kinetic study for PAC

**Fig. 12:**
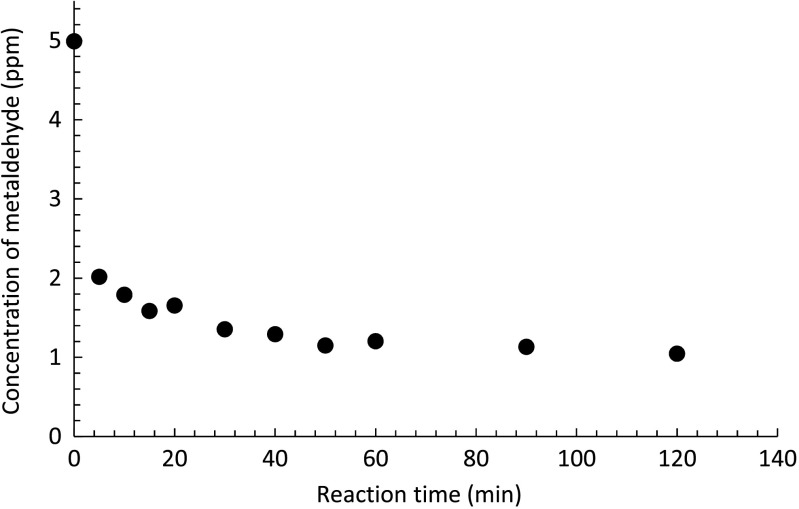
Degradation of 5 ppm metaldehyde with time using 0.1 g PAC under UV-C light with a wavelength of 254 nm

A set of experiments was further performed using 5 ppm metaldehyde and 0.1 g PAC with a 2-h reaction time under UV-C light. Samples were taken at 0, 5, 10, 15, 20, 30, 40, 50, 60, 90, and 120 min. The result is presented in Fig. 12.

At 5 min, the removal of metaldehyde had already achieved 64%, indicating that at the very beginning of the reaction, the adsorption efficiency of PAC was at its highest. For a 2-h reaction time, the total removal of metaldehyde was 81%. The removal of metaldehyde plateaued over time suggesting that PAC was getting saturated gradually.

**Fig. 13:**
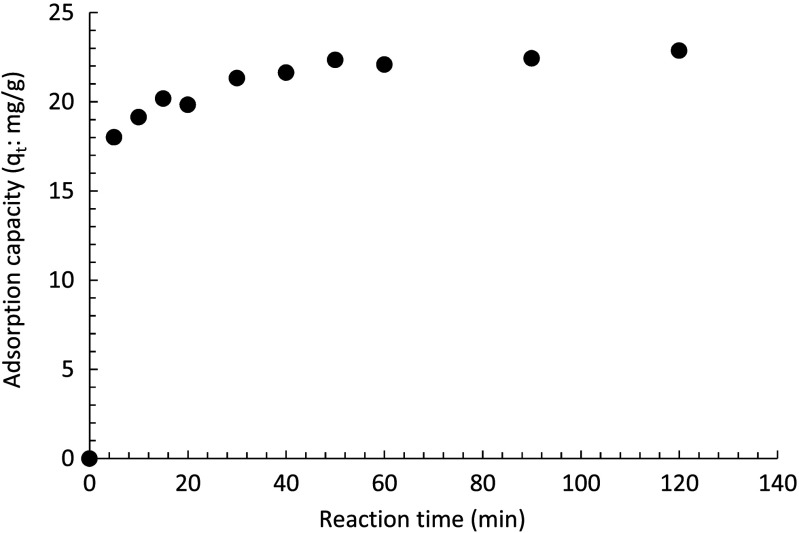
Variation of the PAC adsorption capacity with time under UV-C light with a wavelength of 254 nm

The adsorption capacity (*q*_*t*_) of PAC at different times is presented in Fig. 13. This suggests that PAC adsorbed metaldehyde (*q*_*t5*_ = 18.01 mg g^−1^) at 5 min, almost as soon as the experiment started, with the adsorption capacity at equilibrium (*q*_*e*_) of 22.87 mg g^−1^ at 120 min when the PAC was considered to be saturated. Compared to the experiment of removing 5 ppm metaldehyde using PAC only, the final *q*_*e*_ value is 21.75 mg g^−1^ at 120 min. This implies a slightly higher adsorption capacity of PAC under UV-C light.

To study the adsorption rate and model the adsorption kinetic data, pseudo-first- and pseudo-second-order equations were used as they are the most common kinetic models for adsorption. The pseudo-first-order model, according to Lagergren (1898), assumes that the adsorption rate is proportional to the difference of adsorbate adsorbed at equilibrium (*q*_*e*_) and at time (*q*_*t*_) shown by Eq. (6) (*k*_1_: pseudo-first-order kinetic rate constant).

6 $$ \frac{d{ q}_t}{ d t}={k}_1\left({q}_e-{q}_t\right) $$

Take the log value of each side; Eq. (6) can be linearized:

7 $$ \ln \left({q}_e-{q}_t\right)= \ln {q}_e-{k}_1 t $$

**Fig. 14:**
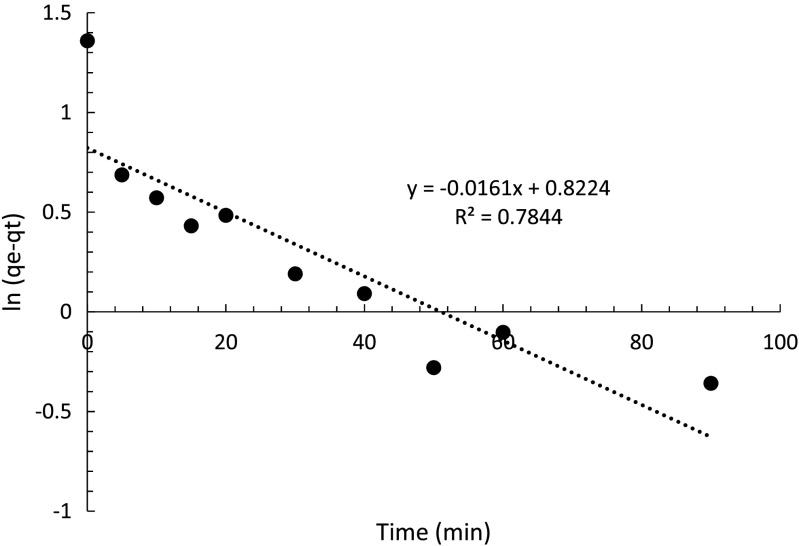
Data fitted to pseudo-first order kinetic model

To fit the data to Eq. (7), ln (*q*_*e*_ − *q*_*t*_) was plotted against time which gives a slope of −*k*_1_ and intercept of ln *q*_*e*_ (Fig. 14).

The *R* ^2^ value is 0.7844, suggesting that the data are not well fitted to the pseudo-first-order model. The intercept is 0.8224 which represents ln *q*_*e*_ and gives a theoretical *q*_*e*_ value of −0.2 mg g^−1^ obtained from the pseudo-first-order model. Nevertheless, this value does not match to the *q*_*e*_ value of 22.87 mg g^−1^ from the experiment. However, compared to the study of Salvestrini et al. (2016) which GAC gave a *k*_1_ value of 0.45 h^−1^ with R^2^ value of 0.87, the adsorption rate constant of PAC here, *k*_1_, is 0.0161 min^−1^ which is 0.966 h^−1^ more than twice higher than that of GAC, suggesting PAC is more efficient regarding the adsorption of metaldehyde.

Since the data do not fit well with the pseudo-first-order model, they are then fit to the pseudo-second-order model. Equation (8) was given by Ho and McKay (1998) in differential form, and *k*_2_ is the pseudo-second order kinetic rate constant.

8 $$ \frac{d{ q}_t}{ d t}={k}_2{\left({q}_e-{q}_t\right)}^2 $$

And it can be integrated to

9 $$ {q}_t=\frac{k_2{ t q}_e^2}{1+{k}_2{ t q}_e} $$

which can be transferred into

10 $$ \frac{t}{q_t}=\frac{1}{k_2{q}_e^2}+\left(\frac{1}{q_e}\right) t $$

**Fig. 15:**
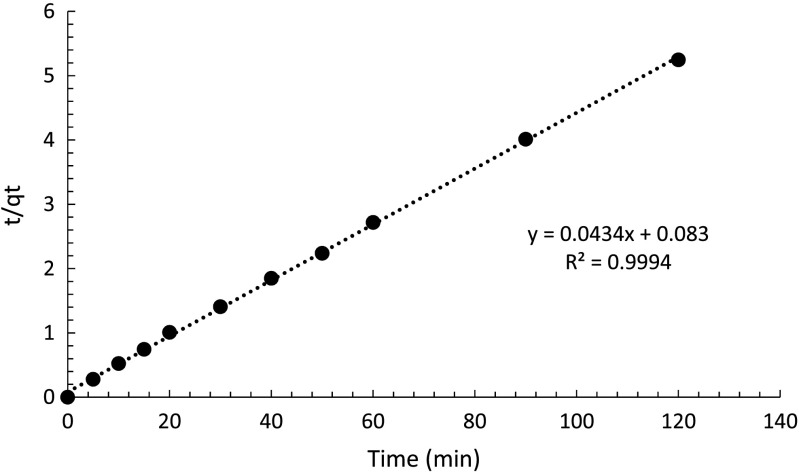
Data fitted to pseudo-second-order kinetic model

To fit the data to Eq. (10), *t*/*q*_*t*_ was plotted against time and from which *q*_*e*_ and *k*_2_ can be calculated (Fig. 15).

The *R* ^2^ value is 0.9994, suggesting that the data are very well fitted to the pseudo-second-order model. The slope of 1/*q*_*e*_ is 0.0434 which gives the theoretical value of *q*_*e*_ of 23.04 mg g^−1^. This value is very close to the value obtained in the experiment, again, confirming that data are well fitted. From the intercept, *k*_2_ can be calculated to be 0.023 g mg^−1^ min^−1^. Compared to the *k*_2_ value obtained from the GAC studied by Salvestrini et al. (2016) of 4.8 × 10^−6^ g μg^−1^ h^−1^ which is 8 × 10^−5^ g mg^−1^ min^−1^, PAC is approximately 288 times more efficient than GAC.

Table 4 compares the key characterization and experimental data of PAC obtained from this project regarding metaldehyde degradation with other studies. It indicates that metaldehyde adsorption is a complex mechanism and the effectiveness and efficiency of metaldehyde adsorption depend very much on the adsorbents. For example, the study of Tao and Fletcher (2013) stated the GAC used has the adsorption capacity of 71 mg g^−1^ which is almost five times higher than the 15 mg g^−1^ capacity of the GAC used by Busquets et al. (2014) while in the surface area the GAC does not differ that much (560 and 500 m^2^ g^−1^, respectively). This suggests that adsorption capacity is not strictly relevant to surface area; more factors such as pore size distribution need to be taken into consideration.

**Table 4:** Comparison of PAC used in this study and other adsorbents regarding metaldehyde degradation

Researches	Materials	Adsorption capacity (mg g^−1^)	Surface area (m^2^ g^−1^)	Efficiency (adsorption rate constant)
This work	PAC	32	962	*k*_2_ = 0.023 g mg^−1^ min^−1^
Busquets et al. (2014)	GAC	15	500	N/A
	Tailored phenolic resin-derived carbon	76	2000	N/A
Salvestrini et al. (2016)	GAC	320	774	*k*_2_ = 8 × 10^−5^ g mg^−1^ min^−1^
Tao and Fletcher (2013)	GAC	71	560	*k*_2_ = 5.8 × 10^−4^ g mg^−1^ min^−1^
Tao and Fletcher (2016)	Macronet (for metaldehyde)	200	402	*k*_1_ = 11.6 × 10^−3^ min^−1^
	Ion-exchange resin (for acetaldehyde)	441	N/A	*k*_2_ = 0.17 g mg^−1^ min^−1^

Moreover, the adsorption capacity of PAC used in this project was 32 mg g^−1^ which is not as high as the GAC used by Tao and Fletcher (2013) and Salvestrini et al. (2016) but it is effective and much more efficient for metaldehyde degradation with a reaction rate 288 times faster than that of Salvestrini et al. (2016) and 40 times faster than that of Tao and Fletcher (2013). This explains that high adsorption capacity does not necessarily mean high adsorption rate. And the adsorption rate is not relative to the surface area as well. The GAC used by Salvestrini et al. (2016) has a high surface area of 774, but the adsorption rate is more than seven times slower than the GAC used by Tao and Fletcher (2013). Therefore, to link characterizations of the adsorbent to adsorption capacity and adsorption rate regarding metaldehyde degradation would require more studies on various characterizations of the adsorbent, such as particle size, pore size distribution, surface analysis, and point of zero charge.

### Fitting adsorption isotherm models for C-doped TiO_2_ (C-1.5) nanocatalyst

**Fig. 16:**
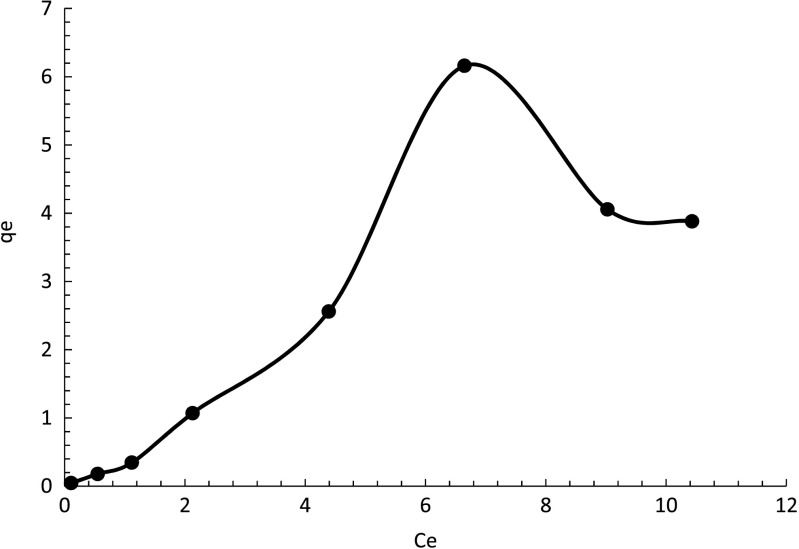
Isotherm of metaldehyde degradation by C-doped TiO_2_ (C-1.5)

The isotherm of metaldehyde degradation by C-doped TiO_2_ (C-1.5) nanocatalyst within 2 h of reaction under UV-C light is shown by Fig. 16. The adsorption capacity in equilibrium (*q*_*e*_) from Eq. (1), *q*_*e*_, has a maximum value of 6.16 mg/g (Kumar et al. 2008).

The isotherm shows that as *C*_*e*_ increases, the adsorption capacity increases until it reaches maximum capacity which corresponded to the highest removal of metaldehyde at 7.5 ppm. The data are also fitted to the Freundlich and Langmuir models.

**Fig. 17:**
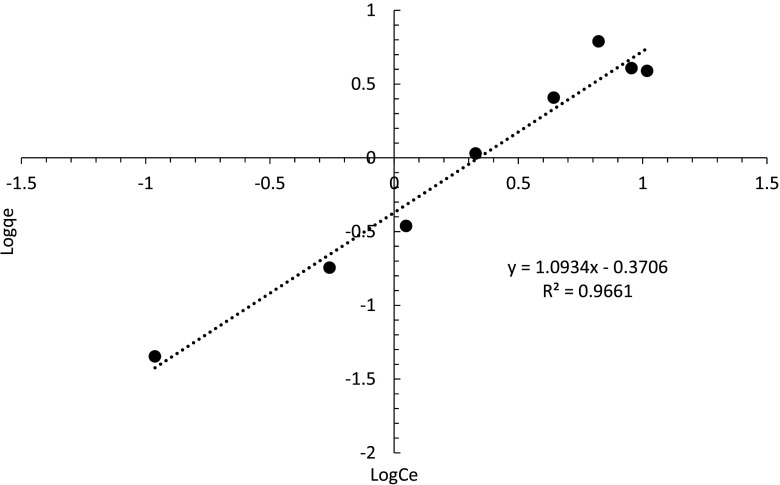
Freundlich model of metaldehyde degradation by C-doped TiO_2_ (C-1.5)

The Freundlich model is plotted (Fig. 17) and data points are fitted by a linear trend line, and the log-log plot gives the intercept of log *K*_*F*_ and slope of 1/*n* (Kumar et al. 2008).

It can be seen from Fig. 17 that the metaldehyde adsorption by C-doped TiO_2_ (C-1.5) obeys the Freundlich model (*R* ^2^ = 0.9661). However, the *K*_*F*_ value obtained is −0.431 L g^−1^ and the *n* value obtained is 0.915. *K*_*F*_, as the indicator of adsorption capacity is a negative value here, which indicates that the material is not suitable for adsorption (Radjenovic and Medunic 2015). This may be explained by the small amount of carbon present in the system.

## Conclusions

Among the four studied materials of PAC, C-doped TiO_2_ (C-1.5), C-40, and C-80 nanocatalysts, PAC was the most effective material for metaldehyde degradation. Within the studied concentration ranges of 0.1, 0.5, 1, 5, and 10 ppm, and reaction time of 2 h, PAC with a loading concentration of 0.2 g L^−1^ showed more significant removal of metaldehyde at low concentrations than higher concentrations. Increasing in the initial concentration of metaldehyde solution did not result in more effective metaldehyde removal. The removal of metaldehyde by PAC decreased as the prepared initial concentration of metaldehyde solution increased. Removal of metaldehyde using PAC by adsorption fits well with the Langmuir kinetic model, giving a *q*_*m*_ value of 32.258 mg g^−1^ and a *K*_*L*_ value of 2.013 L mg^−1^, suggesting that adsorption is favourable for removing metaldehyde. Adsorption of metaldehyde by PAC fits well to pseudo-second-order kinetics and gives a *k*_2_ value of 0.023 g mg^−1^ min^−1^, indicating PAC can remove metaldehyde efficiently in a short period.

Compared to PAC, C-doped TiO_2_ (C-1.5, C-40, and C-80) nanocatalysts were not effective for removing metaldehyde in solution by photocatalysis within the studied concentration range, catalyst loading concentration, light intensity, and reaction time.

The analysis of the effect of UV-C light, and the increasing carbon content of the nanocatalysts, suggests that (1) UV-C light alone does not have any effect on the removal of metaldehyde and (2) increasing carbon content of the nanocatalysts only slightly promotes the degradation of metaldehyde (about 4%). However, PAC alone under dark conditions removed 77% metaldehyde, while it can remove more than 81% under UV-C light. It is considered that metaldehyde is likely to be removed by adsorption of powdered activated carbon. Nevertheless, it would work more effectively under UV-C light. From this study, it is suggested that more parameters such as UV-C light intensity, pH of metaldehyde solution, reaction time, and material loading concentrations can be varied and tested to find out the optimum parameters for metaldehyde degradation.

### Electronic supplementary material

ESM 1 (DOCX 278 kb)

## Appendix

Solid phase extraction method, modified from the UK Environment Agency (2009):

1. Activation of the solvent in the cartridge: 10 mL of HPLC-grade methanol was used to wash the cartridge and the eluate was then discarded. The cartridge must not dry out during the process.
2. Millipore water (2 mL) was added to the cartridge and the eluate was discarded. The cartridge must not dry out during the process.
3. Sample solution (1 mL) was added to the cartridge. The eluate was discarded.
4. The cartridge was then left for 15 min to ensure that metaldehyde was completely absorbed by the solvent.
5. Another 2 mL of Millipore water was added to the cartridge. The eluate was discarded and the cartridge was dried by passing air through it via a vacuum pump for at least 45 min to ensure the cartridge was completely dry.
6. After the cartridge was dried, a suitable vial was placed inside the SPE vacuum manifold.
7. Dichloromethane (DCM) 3 mL was added to the cartridge and the eluate was collect in the vial.
8. Residual DCM was collected in the vial by passing air through the cartridge.
9. The vial was then removed from the SPE vacuum manifold, and the 3-mL DCM equate was reduced to 1 mL by evaporation with nitrogen gas.
10. The 1 mL of DCM was then transferred to a suitable Perkin Elmer GC-MS vial by a glass micropipette and was ready to be analysed.
