# Supplementary material for: Degradation of metaldehyde in water by nanoparticle catalysts and powdered activated carbon
Source: Environ Sci Pollut Res Int. 2017 Jun 14;24(21):17861–73. doi: 10.1007/s11356-017-9249-1 (PMC5533860; doi:10.1007/s11356-017-9249-1)
Supplement: Supplementary file 1 — (DOCX 278 kb) [file 11356_2017_9249_MOESM1_ESM.docx]

Article

Oxidation and adsorption of metaldehyde in water by nanoparticle catalysts and powdered activated carbon

Journal name: Environmental Science and Pollution Research

Zhuojun Li ^1^, Jong Kyu Kim^2^, Vrushali Chaudhari ^3^, Suseeladevi Mayadevi ^3^, and Luiza C. Campos ^1^*

^1^ Department of Civil, Environmental and Geomatic Engineering, University College London, Gower Street, London, WC1E 6BT, UK ; Emails : zhuojun.li.09@ucl.ac.uk; l.campos@ucl.ac.uk

^2^ Kyungnam University, Gyeongsangnam-do Massanhappo-gu South Gyeongsang Daehakro 7, Changwon, South Korea; Email: jongkim@kyungnam.ac.kr

^3^ Chemical Engineering & Process Development Division, CSIR-National Chemical Laboratory, Pune, Maharashtra 411 008, India; Emails: s.mayadevi@ncl.res.in; 17vrushali@gmail.com

***** Author to whom correspondence should be addressed; Email: l.campos@ucl.ac.uk (L.C.C.); Tel.: +44-207-679-4162 (L.C.C)

# **Supplementary Materials:**

**A**

**B**

**C**

**E**

**D**

**Fig.S1** Pure DCM analysis prior to metaldehyde standard calibration solution: A (first injection) to E (last injection) show 5 injections of the same DCM sample. B shows that new peak at 7.62 min formed at this injection, and D shows that another new peak at 7.90 min formed at this injection.

During the time of analysis by GC-MS, some DCM may have decomposed into these components and therefore were detected, showing these peaks at 7.64 min, 7.84 min, 7.90 min, and 8.14 min. This also correlated to pure DCM analysis prior to sample analysis by GC-MS before the injection of samples. As Figure 1 demonstrates, pure DCM was injected five times, and the first injection only detected one peak of DCM at 9.71 min. However, after that, all consequent injections showed additional peaks around 7.64 min, 7.90 min, and 9.72 min. This was highly likely due to some DCM that started to decompose from the heat of the GC-MS when running at with the injection temperature of 180 ºC and the oven temperature at 150 ºC.

**Table S1.** Parameters used for detection of metaldehyde by GC-MS [1]

| **Parameters** | **Conditions** |
| --- | --- |
| **Carrier gas** | Helium, 30 mL s^-1^ |
| **Column** | Rxi-5ms, 30 m × 0.25 mm diameter, 1.0 µm film thickness |
| **Injection volume** | 1 µL (pulsed split-less injection) |
| **Injection temperature** | 180 °C |
| **Temperature programme** | Oven  Initial temperature 100 °C for 1 min, then 5 °C min^-1^ to 150 °C, hold time for 1 min. |
| **Purge flow** | 50 mL min^-1^ |
| **Purge time** | 1 min |
| **Total flow** | 52.7 mL min^-1^ |
| **Solvent delay of MS** | 4 min |

**Table S2.** Recovery rate

**Table S2 (A).** Recovery rate of metaldehyde samples at 0 hour (PAC experiment)

| **Prepared concentrations (ppm)** | **Calculated concentrations (ppm)** | **Recovery rate (%)** |
| --- | --- | --- |
| **0.1** | 0.091 | 91 |
| **0.5** | 0.466 | 93.2 |
| **1** | 0.96 | 96 |
| **5** | 4.857 | 97.14 |
| **10** | 9.76 | 97.6 |

**Table S2 (B).** Recovery rate of metaldehyde samples at 0 hour (C-doped TiO_2_ nanocatalyst experiment)

| **Prepared concentrations (ppm)** | **Calculated concentrations (ppm)** | **Recovery rate (%)** |
| --- | --- | --- |
| **0.1** | 0.118 | 118 |
| **0.5** | 0.586 | 117.2 |
| **1** | 1.187 | 118.7 |
| **2.5** | 2.347 | 93.88 |
| **5** | 4.904 | 98.08 |
| **7.5** | 7.88 | 105.07 |
| **10** | 9.836 | 98.36 |
| **12** | 11.208 | 93.4 |

**Table S2 (C).** Recovery rate of metaldehyde samples of 5 ppm for different treatments

| **Treatment** | **Calculated concentrations (ppm)** | **Recovery rate (%)** |
| --- | --- | --- |
| **C-1.5 only** | 5.325 | 106.5 |
| **UV-C only** | 4.789 | 95.78 |
| **C-1.5 + UV-C** | 4.901 | 98.02 |
| **C-40 + UV-C** | 5.033 | 100.66 |
| **C-80 + UV-C** | 5.375 | 107.5 |
| **PAC + UV-C** | 5.618 | 112.36 |
| **PAC only** | 5.663 | 113.26 |

**
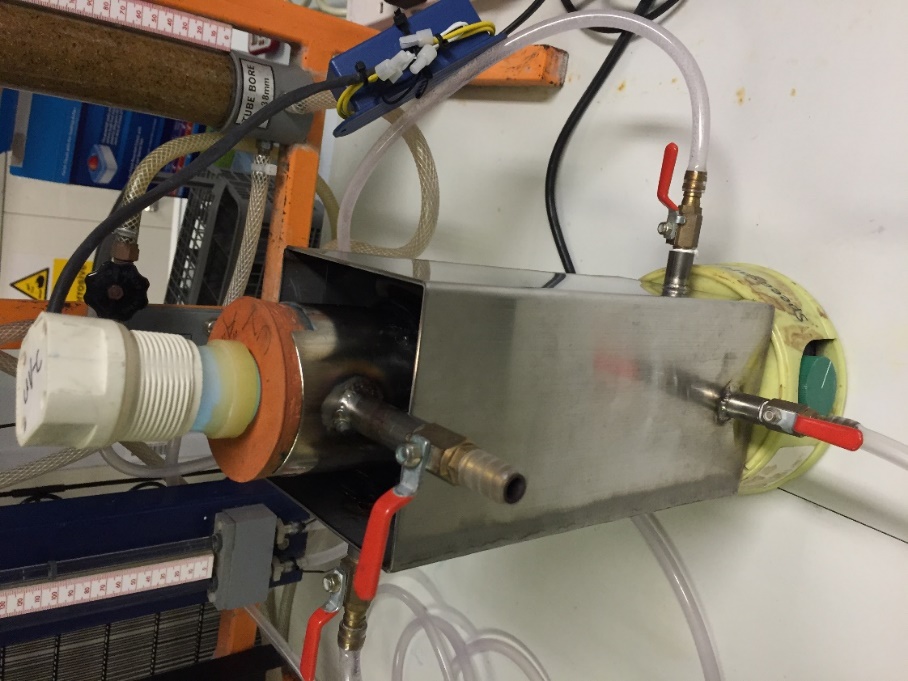
Fig.S2** Batch photoreactor system


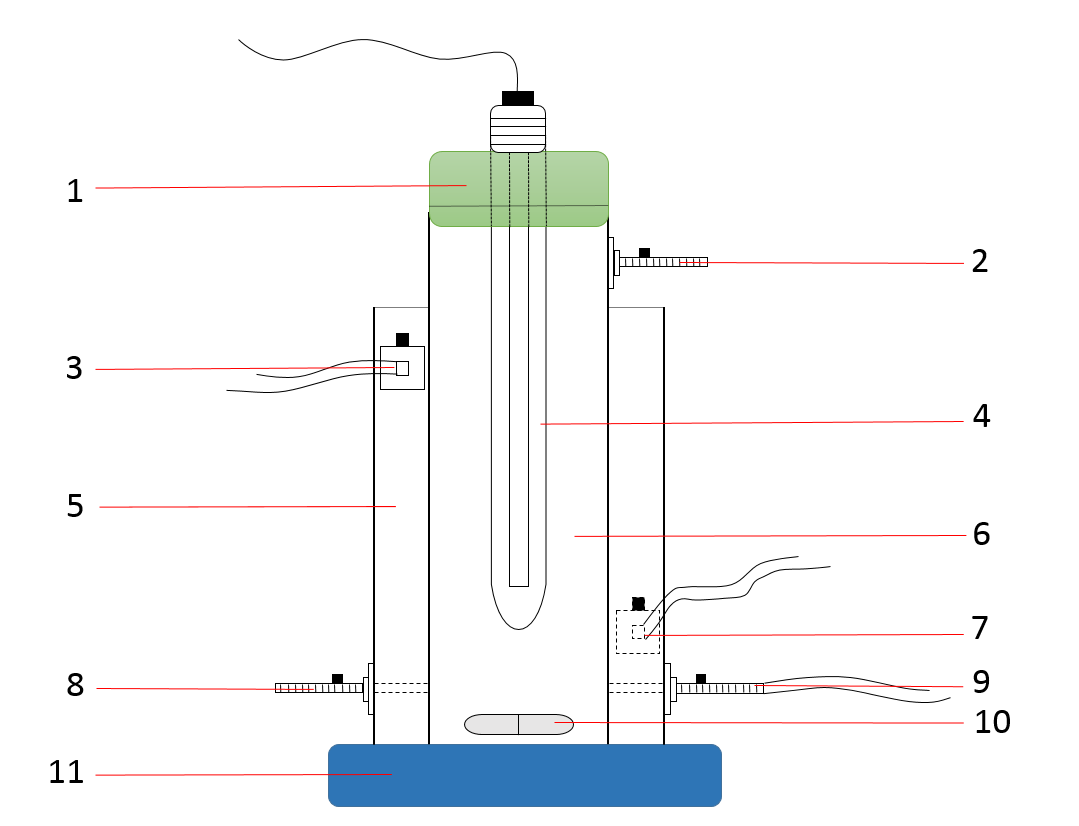
**Fig.S3** Side view of the batch photoreactor system configuration

1. Rubber bung fitting at the top of the reactor, holding the UV-C lamp
2. Closed top valve connecting to the reactor
3. Opened valve on the side of the water cooling jacket connecting outflow of cooling water
4. UV-C light in the quartz sleeve
5. Water cooling jacket
6. Central photoreactor
7. Opened valve on the other side of the water cooling jacket connecting inflow of cooling water connecting from water tap
8. Closed bottom valve connecting to the reactor
9. Opened bottom valve connecting to air supply
10. Magnetic stirrer
11. Magnetic stirrer controller
